# Professor Arloing

**Published:** 1911-04-08

**Authors:** 


The death is announced from Lyons of Professor Arloing, who was Professor of Experimental Medicine at the University of that town, as well as Director of the Veterinary College. Born at Cusset in 1846, he became the pupil of Chaveau at the Veterinary College of Lyons, and later his chief laboratory assistant. In 1878, on the creation of the Faculty of Medicine of Lyons he took his doctor's degree, and was then appointed to the Chair of Physiology in the Faculty of Science. This he eventually vacated on being appointed to the posts he held at the time of his death. He was the author of numerous scientific papers connected with physiology, bacteriology, and experimental medicine, as well as of several books on comparative anatomy, bacteriology, and medicine, as the result of which he was elected an Associate of the Academy of Medicine and correspondent of the Institute.

